# Age-related alterations in trunk extensor force control during isometric and isokinetic contractions

**DOI:** 10.1038/s41598-026-41572-6

**Published:** 2026-03-12

**Authors:** Martina Parrella, Michail Arvanitidis, Riccardo Borzuola, David Jiménez-Grande, Andrea Macaluso, Deborah Falla

**Affiliations:** 1 Department of Movement, Human and Health Sciences, Università degli Studi di Roma “Foro Italico”, Rome, Italy; 2 Centre of Precision Rehabilitation for Spinal Pain (CPR Spine), School of Sport, Exercise and Rehabilitation Sciences, College of Life and Environmental Sciences, University of Birmingham, Birmingham, UK

**Keywords:** Medical research, Neuroscience, Physiology

## Abstract

Research on age-related changes in trunk extensor force control is currently limited, and the underlying neuromuscular mechanisms remain largely unexplored. To address this, we examined the relationship between oscillations in lumbar erector spinae (LES) activity and torque fluctuations in 20 young and 20 older adults during isometric and isokinetic (concentric) trunk extension contractions at 25% and 50% of maximal voluntary contraction (MVC). High-density surface electromyography (HDsEMG) signals were recorded bilaterally from the LES using 64-electrode grids. Torque steadiness was quantified using the coefficient of variation (CoV) of torque. Coherence analysis in the δ band (0–5 Hz) was applied between filtered interference HDsEMG and torque signals. Topographical maps were also generated to assess regional differences in HDsEMG-torque coherence. Older individuals exhibited greater torque CoV than young adults during both isometric (+ 23.03%, *p* < 0.001) and isokinetic (+ 72.62%, *p* < 0.001) contractions, with a larger between-group difference at 25% MVC for isokinetic contractions (Group × Torque interaction; *p* = 0.007). At this intensity, the older group also showed reduced HDsEMG-torque coherence (Group × Torque interaction; *p* = 0.004). During isometric contractions, coherence magnitude was similar across groups (*p* > 0.05), but older adults exhibited higher coherence in more cranial and medial LES regions (*p* = 0.005 and *p* = 0.001, respectively). Older individuals exhibited the greatest impairment in force steadiness during low-intensity isokinetic contractions. Distinct neuromuscular patterns, possibly influencing force control, emerged depending on contraction type.

## Introduction

The force a muscle produces during voluntary movements depends on the number of motor units recruited and their discharge rates^1^. However, even when a person attempts to apply a constant force, the output is not perfectly steady but rather, it fluctuates around an average value^2^. These fluctuations are commonly quantified using magnitude-based measures, such as the standard deviation and coefficient of variation, which provide an index of force/torque steadiness^2^.

Several studies have reported increased force fluctuations in older individuals during both isometric^3–5^ and concentric or eccentric contractions^6–8^ of the upper and lower limb muscles. From a functional perspective, this suboptimal control of muscle force may contribute to a decline in physical performance, affecting a wide range of balance and mobility tasks^9–11^. Given these functional consequences, the neural mechanisms underlying force fluctuations have been a longstanding focus of research in ageing^12^.

Low-frequency components (< 10 Hz) of the neural drive to the muscle (i.e., the effective neural drive) are mainly responsible for force generation, as they reflect the common synaptic input to the motoneuron pool^13–15^. This common input determines the neural command required for optimal force generation, while oscillations within this input challenge the stability of the force output^16^. Consequently, fluctuations in the effective neural drive determine force variability^16^. Accordingly, Castronovo et al.^17^, using high-density surface electromyography (HDsEMG) decomposition analysis, reported that reduced force steadiness in older individuals when performing submaximal contractions of the dorsiflexor muscles was associated with increased fluctuations in the common synaptic input to motoneurons. However, the decomposition of HDsEMG signals remains challenging for some muscles, including the lumbar erector spinae. As an alternative, examining the surface EMG (sEMG)/force relationship provides a useful approach to better understand the interaction between muscle activity and force production. Using this approach, previous studies have demonstrated that the low-frequency force fluctuations are correlated with the low-frequency components of the rectified interference sEMG^18,19^. In addition, combining HDsEMG, which offers higher spatial sampling resolution than traditional sEMG, with principal component analysis (PCA), has been shown to enhance sEMG-based force estimation^20,21^. PCA is a dimensionality-reduction technique that captures the common variability across multiple HDsEMG signals, selecting the most relevant components to generate a unique signal that explains most of the variance in the exerted force^21^. In addition, since HDsEMG recordings can be used to generate topographical maps of the amplitude of muscle activity, oscillations in HDsEMG and torque signals can also be assessed in the spatial domain using maps that quantify the relationship between these two signals across the regions covered by the electrode grid. This approach helps determine whether specific muscle regions contribute more to the exerted torque than others^22^. This approach has previously been used to assess force control of the trunk extensor muscles during both isometric and dynamic contractions in individuals with chronic low back pain^22–24^. However, this methodology has never been applied in older adults, where it could provide valuable insights into the effects of ageing on trunk extensor function.

The trunk is extremely relevant to investigate since it plays a fundamental role in activities of daily living by providing proximal stability for distal mobility^25^. In particular, the trunk extensor muscles are the primary stabilisers of the spine, counteracting the anterior flexion moments imposed by gravity, which is essential for maintaining an upright posture^26,27^. Trunk stability, as measured by an unstable sitting posturographic test, has been shown to be significantly associated with whole-body dynamic balance^28^. In addition, trunk extensor torque steadiness showed the strongest association with vertebral fracture occurrence in older women among several trunk muscle parameters, including peak torque, rate of torque development, and endurance^29^.

Previous research has shown that older adults exhibit greater force fluctuations than younger individuals during a fatiguing task at 30% of maximal voluntary isometric force^30^ and a 15-s steadiness task at 10% of peak torque^31^. In addition, older individuals with hyperkyphosis exhibited altered position and force sense (i.e., the ability to accurately reproduce a specific target position and force) of the trunk extensors, suggesting impaired proprioception^32^. Notably, Forestieri Faccio et al.^25^ found that torque steadiness was overall greater in the trunk flexor muscles than in the trunk extensors of older individuals, suggesting that the force control of the trunk extensor muscles may be more affected by age. However, evidence on how ageing affects trunk extensor force control remains limited, and all of the studies mentioned above have focused exclusively on isometric contractions, whereas dynamic contractions better resemble movements of daily activities^33^. Moreover, the neuromuscular mechanisms underlying age-related alterations in force control in these muscles have yet to be elucidated.

This study aims to quantify the relationship between oscillations in lumbar erector spinae (LES) HDsEMG amplitude and torque fluctuations in the frequency domain in young and older adults during isometric and concentric trunk extension contractions. Additionally, regional differences in HDsEMG-torque coherence of the LES are analysed during both types of contractions. We hypothesised that older individuals would exhibit greater torque fluctuations than younger individuals during both isometric and dynamic contractions, with this difference being more pronounced in dynamic contractions^12^. Furthermore, given the age-related alterations in motor unit properties and neural input to motor neurons^12^, we hypothesised that older individuals would show lower coherence between HDsEMG and torque, along with a different regional distribution of LES HDsEMG-torque coherence^30^, which would suggest altered coupling between neural input to the target muscles and mechanical output.

## Methods

### Design and setting

This study employed a cross-sectional design and was approved by the Ethics Committee of the University of Birmingham, United Kingdom (ERN_3410). All experimental procedures were conducted in accordance with the Declaration of Helsinki, and the study is reported following the STROBE guidelines^34^. Data collection took place between March and June 2025 at the Centre of Precision Rehabilitation for Spinal Pain (CPR Spine), University of Birmingham, United Kingdom. Participants attended a single experimental session lasting approximately 1.5 h, and provided written informed consent prior to participation. They were encouraged to raise any questions or concerns regarding the study before providing consent.

### Participants

Twenty older volunteers and twenty younger volunteers were enrolled in the study. The sample size was determined a priori based on a statistical power analysis (G*Power statistical software v.3.1.9.4) for a mixed-model ANOVA (within-between interaction) with α = 0.05, power (1–β err prob) = 0.80, and effect size = 0.25. These parameters were based on previous similar studies investigating torque steadiness and HDsEMG-torque coherence, which reported small to moderate effect sizes^22,30^.

Young participants (age range: 18–35 years) were included if they did not have a history of neurological or orthopaedic disorders, while older participants (age range: 65–80 years) were included if they met the criteria to be defined as “medically stable” for exercise studies^35^. In addition, both young and older volunteers were excluded from the study if they had a history of chronic low back pain which warranted them to seek care from a healthcare practitioner, current low back pain, lumbar radiculopathy, spinal surgery or spinal deformities (i.e., scoliosis, spondylolisthesis, spondylolysis)^30^. Participants were recruited from the local Birmingham area and the University of Birmingham student and staff communities via social media advertisements, posted information leaflets, and word of mouth. In addition, older participants were recruited through the *Birmingham 1000 Elders* program, a registry of healthy older adults maintained by the university for participation in research studies.

### Functional assessment

Before starting the session, blood pressure was measured in older participants to ensure they were fit to participate. Participants were excluded from testing if their resting diastolic blood pressure was ≥ 100 mmHg and/or their resting systolic blood pressure was ≥ 160 mmHg^36^. If blood pressure values were within the acceptable range, a functional assessment was performed using the Berg Balance Scale (BBS). The BBS consists of fourteen functional tasks of increasing difficulty, ranging from quiet stance, sit-to-stand, weight shifting, and reaching, to turning in place, tandem stance, and single-leg stance^37,38^. Each task was scored on a 5-point scale from 0 (unable to perform) to 4 (performed independently) according to standardised criteria. The maximum total score was 56 points, with higher scores indicating better balance and the absence of detectable balance difficulties. Participants were allowed to rest as needed between tasks.

### Questionnaires

All participants were asked to complete a questionnaire to assess their physical activity level. Specifically, young adults completed the Baecke Physical Activity Questionnaire (BPAQ), a validated self-report instrument consisting of sixteen questions that assess physical activity during a typical week over the past year across three domains: (1) work (eight questions), (2) sport/exercise (four questions), and (3) leisure-time non-sport activity (four questions)^39,40^. In the sport/exercise domain, participants answered three additional questions regarding the type of activity, the average number of hours per week, and the number of months per year they engaged in that activity. Except for questions about main occupation and type of sport/exercise that had three response options (i.e., low, moderate, or high intensity/activity), all other items were rated on a five-point Likert scale, ranging from never to always/very often. Each domain yielded a score between 1 and 5, which were then summed to produce a total physical activity score ranging from 3 (minimum) to 15 (maximum).

Since the original version of the BPAQ has been shown to be less reliable in elderly populations^41^, an adapted version, the Modified BPAQ, was used for the older group^42^. This version assesses three domains: (1) household activities, (2) sport/exercise, and (3) leisure-time activities. The household domain consists of ten questions, each with four to five response options. For the sport/exercise and leisure-time domains, participants reported the type of activity (up to two sports and up to six leisure-time activities), specifying the intensity, hours per week, and months per year for each activity. Scores from the household, sport/exercise, and leisure-time domains were summed to produce a continuous, unitless total physical activity score.

The total scores from both the BPAQ and the Modified BPAQ were divided into tertiles based on group-specific cutoffs to classify participants as having low, moderate, or high habitual physical activity, in line with previous studies^41,43,44^.

### Dynamometry

**Fig. 1:**
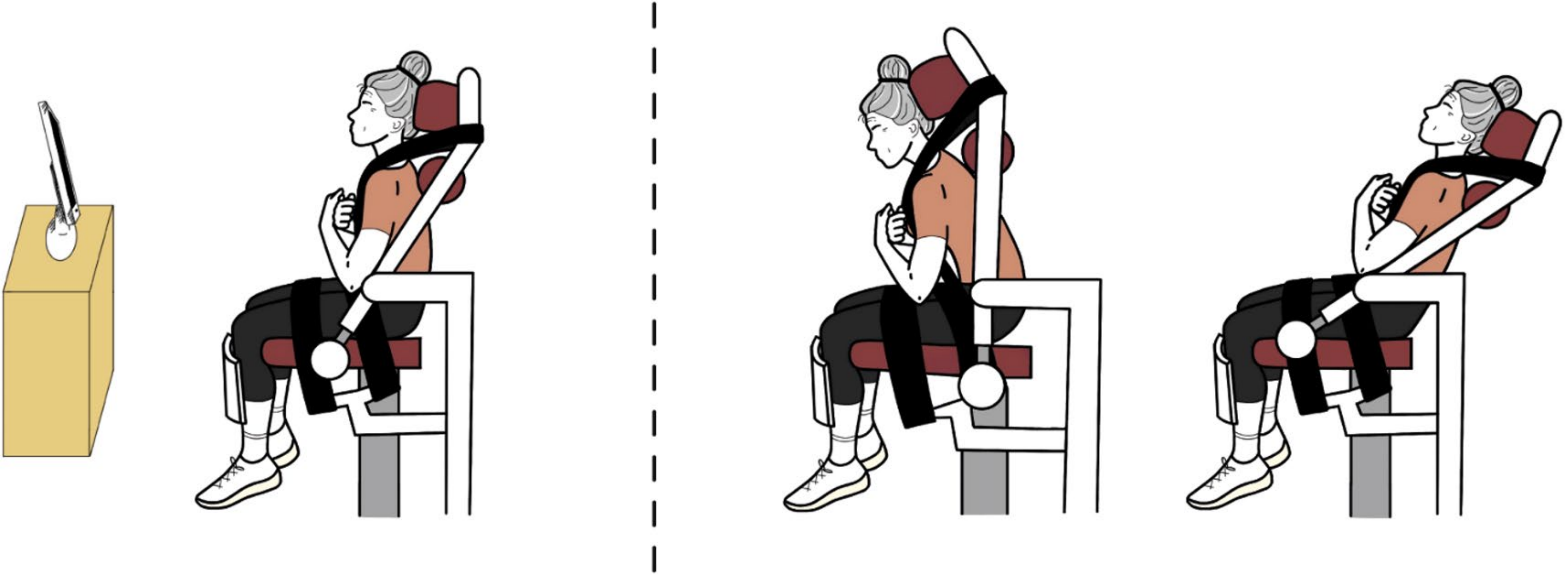
Illustration of the experimental setup. The participant is seated on the Biodex chair during **(a)** isometric trunk extension tasks, with the hip-to-trunk angle set at 90°, and during isokinetic trunk extension tasks, starting from **(b)** 30° of hip-to-trunk flexion and ending at **(c)** 20° of hip-to-trunk extension.

The torque exerted by participants during maximal voluntary contractions (MVCs) and submaximal torque steadiness tasks was measured using an isokinetic dynamometer (System 3 Pro, Biodex Medical Systems, New York). All contractions were performed on the Biodex Dual Position Back Extension/Flexion Attachment, with participants’ knees flexed at 90° and their feet positioned parallel to the floor at a distance equal to the inter-acromial distance (Fig. 1). The front of the seat was tilted approximately 15° clockwise, and the height of the chair was adjusted so that the rotational axis of the dynamometer was aligned bilaterally with the anterior superior iliac spines of participants. This position, referred to as the compressed isolated lumbar position, was selected to maximise the contribution of LES muscle to the resultant torque^22^. Participants’ upper trunk, thighs, and pelvis were also securely strapped to the seat to minimise compensatory movements during the contractions, and a specific attachment was used to reduce involvement of the knee muscles.

For the isometric trunk extension contractions, the dynamometer was locked at a hip-to-trunk angle of 90° (Fig. 1a). In contrast, concentric trunk extension contractions were performed in isokinetic mode over a total range of motion of 50°, starting at 30° of hip-to-trunk flexion (Fig. 1b) and ending at 20° of hip-to-trunk extension (Fig. 1c). This setup was chosen in order to better isolate lumbar motion and minimise compensatory movements from the lower extremities^24^. The angular velocity for the extension phase was set at 10°·s⁻¹, while it was set at 60°·s⁻¹ when returning to the starting position. However, the return phase was performed passively by the researcher, who repositioned the chair after each repetition. The same setup was used for both MVCs and submaximal tasks for each type of contraction (isometric or isokinetic).

Torque signals were sampled at 2048 Hz, digitised at the source with a 16-bit A/D converter (Quattrocento, OT Bioelettronica, Torino, Italy) and synchronised with the electromyogram via an auxiliary input. Recordings were performed using the OTBiolab+ software.

### HDsEMG

**Fig. 2:**
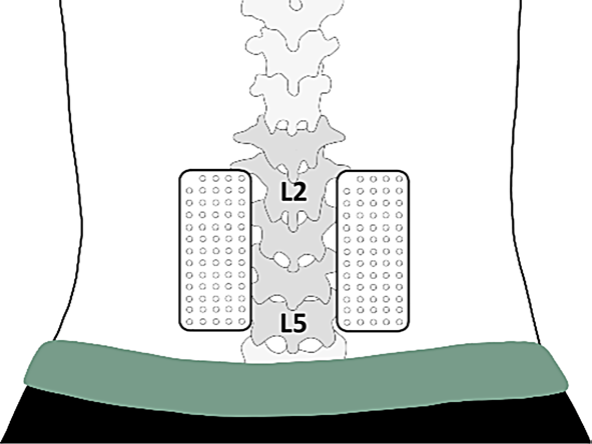
Grids of electrodes placed over the right and left LES. The L5 and L2 vertebral landmarks are indicated to illustrate electrode placement.

The CEDE checklist was used to ensure accurate reporting of the acquisition and processing of EMG data^45^. HDsEMG signals were recorded bilaterally in monopolar mode from the LES during both MVCs and submaximal contractions, using adhesive grids of 64 equally spaced electrodes (GR08MM1305, gold-coated, 1 mm diameter, 8 mm interelectrode distance; OT Bioelettronica, Turin, Italy). Each grid consisted of 13 rows and 5 columns of electrodes with a missing electrode in the upper left corner. Before electrode placement, a double-sided adhesive foam (FOA08MM1305; OT Bioelettronica) was applied to the grids, and the electrode cavities were filled with conductive paste (AC-CREAM; SPES Medica, Genoa, Italy) to ensure proper electrode-skin contact. After identifying the perimeter of the right and left LES by manual palpation, the participant’s skin was shaved, gently abraded using abrasive paste and cleaned with water to ensure the best conductivity of HDsEMG signals. As described by Falla et al.^46^, the two grids were placed ~ 2 cm lateral to the L5 spinous process mid-point, covering the low back approximately from L5 to L2 on both the right and left sides (Fig. 2). To reduce electrical noise and improve signal quality, three reference electrodes were placed on the participant: one over a lumbar or thoracic vertebra and one on each wrist.

HDsEMG signals were amplified (x150), sampled at 2048 Hz, band-pass filtered (10–500 Hz, first order, -3 dB), and digitised with a 16-bit A/D converter (Quattrocento, OT Bioelettronica, Torino, Italy).

### Testing protocol

Participants were first familiarised with the dynamometer to warm up their trunk extensor muscles. The testing protocol consisted of two parts: isometric contractions followed by isokinetic concentric contractions.

For the isometric contractions, the warm-up consisted of three sets of eight low-to-moderate intensity isometric contractions, with one minute of rest between sets. Participants then performed three isometric MVCs, separated by 2 min of rest. If necessary, additional MVCs were performed until consistent values were achieved. The highest maximal voluntary torque obtained from the consistent MVCs was used to determine the target torque for the submaximal tasks. After completing a few practice trials, participants performed two sustained isometric trunk extension contractions at 25% MVC (2.5-s ramp-up, 30-s hold, 2.5-s ramp-down) and two at 50% MVC (5-s ramp-up, 15-s hold, 5-s ramp-down). A rest period of 30 s was provided between contractions at 25% MVC and 1 min between contractions at 50% MVC. The order of torque levels was randomised, with a 2-min rest period separating the two intensities. After the submaximal tasks, the isometric MVC test was performed again to rule out the presence of muscle fatigue.

For the isokinetic contractions, the protocol followed essentially the same structure as the protocol for isometric testing. The warm-up consisted of three sets of five low-to-moderate intensity contractions performed across the full 50° range of motion (from 30° hip-to-trunk flexion to 20° extension), with one minute of rest between sets. Participants then performed the isokinetic MVCs, separated by 2 min of rest, and the highest torque obtained was used for the submaximal tasks. After a few practice trials, participants performed four submaximal concentric trunk extension contractions at 25% MVC and four at 50% MVC. With an angular velocity of 10°·s⁻¹ over a 50° range of motion, each contraction lasted 5 s. During these tasks, unlike the isometric contractions, no ramped contractions were included. Instead, participants were instructed to maintain their exerted torque, displayed as a bar, aligned with a single target line (25% or 50% MVC) throughout the range of motion. Rest periods of 15 s and 30 s were provided between contractions at 25% and 50% MVC, respectively, with the order of intensities randomised and separated by a 2-min rest period. An additional isokinetic MVC test was performed after the submaximal tasks again to rule out the presence of muscle fatigue.

During all submaximal contractions (both isometric and isokinetic), participants received real-time visual feedback of the target torque on a computer monitor positioned 1.5 m in front of them (Fig. 1). They were instructed to reach the specified % MVC target (25% or 50%) and maintain their torque as steadily as possible for the duration of the task. Instructions were provided prior to the contractions. Participants received verbal encouragement to exert maximal effort during the MVCs, whereas no feedback was given during the submaximal tasks, and the testing environment was kept quiet.

## Data analysis

### PCA

PCA was applied to reduce the high dimensionality of the HDsEMG data, which can introduce redundant or correlated information in the recorded signals, such as noise, artefacts, or channels with low muscle activity^47^. In particular, PCA was implemented to reduce the large set of correlated sEMG signals into a smaller subset of uncorrelated variables defined principal components (PCs)^22^. According to Naik et al. ^47^, PCs capturing more than 80% of the data variance are typically retained. In the present study, this threshold was set at 85% of total variance.

From each of the two HDsEMG grids (i.e., on the right and left LES muscles), 59 adjacent bipolar channels were derived from the 64 monopolar signals, resulting in 12 longitudinal bipolar recordings per column, except for the far-left column, which contained 11 electrode pairs due to the missing electrode. Prior to coherence analysis, the bipolar HDsEMG channels were processed as follows^21,22^: (1) high-pass filtering at 10 Hz; (2) selection of the most informative subset of PCs obtained by applying PCA to the 59 differential HDsEMG signals in the time domain; (3) full-wave rectification and averaging of the selected PCs to generate a single time-domain signal for each muscle; (4) additional low-pass filtering at 10 Hz to capture slow frequency fluctuations in motor unit firing rate/recruitment^14^, (5) smoothing with a first-order Savitzky-Golay filter; and (6) removal of the DC (zero-frequency) components. These processing procedures resulted in the Final Signal Envelope, obtained by applying PCA to the HDsEMG grid, which contained the relevant force-related information (i.e., low-frequency components of the HDsEMG data).

After extracting the Final Signal Envelope, coherence analysis was performed to quantify the similarity between the HDsEMG envelope and the torque signals. This analysis was conducted for all submaximal contractions (both isometric and isokinetic), with similarity calculated for each repetition and then averaged to yield a single coherence value at a given torque level.

### Coherence analysis

Coherence analysis was performed to indirectly estimate the strength of common rhythmic synaptic inputs across the motor unit pool and to examine their association with torque^22^. The coherence between HDsEMG envelope and torque signals was computed using the magnitude-squared coherence (MSC) method with a 1-s Hamming window and 50% overlap, as previously reported^48^. In particular, the Final Signal Envelope obtained from the PCA analysis was correlated with the torque signal in the frequency domain. The MSC approach is frequently used to quantify the linear correlation between two signals in the frequency domain, ranging from 0 (no correlation) to 1 (perfect correlation). In this study, MSC was computed using the *mscohere* function of the MATLAB Signal Processing Toolbox.

Coherence analysis focused exclusively on the δ band (0–5 Hz), as this frequency range is most relevant for force generation^16^. Coherence within this band was quantified by integrating the coherence estimates across the δ frequency range. To enable statistical comparison, coherence estimates (C) were converted to Fisher’s z values (FZ), as indicated in **Eq. (1)**^48^. Since coherence can be influenced by crosstalk commonly contaminating sEMG recordings, the bias was empirically determined and removed. This bias was defined as the maximum value of the coherence profile at 250 Hz, a frequency range where no significant correlated activity is expected^48^.

1 $$\:FZ=atanh\:\left(\sqrt{C}\right)\:-bias$$

Equation 1 Conversion of coherence values (C) into Fisher’s z-scores (FZ).

Additionally, topographical maps of coherence were generated, as previously described^22,24^. In these maps, each of the 59 bipolar recordings within each HDsEMG grid was individually assessed for coherence with the filtered torque signal. This procedure yielded 59 coherence values in the δ band per grid, which were then used to construct the topographical coherence maps. These maps were then normalised to the maximum coherence observed at each torque level (25% or 50% MVC). The centroid of coherence was calculated to determine its spatial location along the medial-lateral (x-axis) and cranial-caudal (y-axis) directions. This approach allowed us to assess whether specific muscle regions contributed more to torque generation.

Coherence variables described above were extracted from the steady portion of the submaximal contractions using a non-overlapping 0.5 s sliding averaging window with a custom MATLAB script (MATLAB 2022b, Mathworks Inc., Natick, MA, USA). The script plotted the torque signal of each participant, and the steady portion of each contraction was identified to determine the start and end points for analysis. In particular, for the isometric contractions, the first and last second of the steady portion of each repetition were removed, whereas for the isokinetic contractions, the first and last 0.5 s were removed due to their shorter duration. These initial and final portions were excluded because they typically contain overshoot or undershoot of the target force^23^. All variables were computed separately for each repetition and then averaged to obtain a single value for each torque level within each contraction condition (isometric or isokinetic).

### Root mean square

For these calculations, the 59 adjacent bipolar channels from each grid were processed differently from the PCA procedure described above, in order to compute the root mean square (RMS) of the HDsEMG signals. Prior to these analyses, HDsEMG signals were band-pass filtered (10–350 Hz, second-order, zero-lag Butterworth)^49^. The signal quality of each channel was also visually inspected, and channels considered of low quality (e.g., due to noise or artifacts) were excluded. As the removal rate was below 15% in this study, RMS values could be estimated reliably from all submaximal trunk extension tasks, including both isometric and isokinetic contractions^50^.

RMS values were computed for each bipolar channel of both HDsEMG grids. The 59 RMS values obtained from each grid were then averaged to yield a single representative value of the global myoelectric activity (RMS_mean_) for each muscle during each task. Specifically, as with the coherence variables, RMS_mean_ values were extracted from the steady portion of the submaximal contractions, and were computed separately for each repetition. The values were then averaged to obtain a single value for each torque level within each contraction condition (isometric or isokinetic).

To allow statistical comparison between participants, the RMS_mean_ values were normalised to the maximum RMS (RMS_max_) obtained during the MVC tests, resulting in normalised RMS values (RMS_norm_). In particular, RMS_mean_ values from the submaximal isometric tasks were normalised to the RMS_max_ recorded during the highest isometric MVC, whereas RMS_mean_ values from the submaximal isokinetic tasks were normalised to the RMS_max_ obtained during the highest isokinetic MVC. A 200-ms window around the peak torque was used to extract the RMS_max_ values during the MVC of each type of contraction.

### Torque signal analysis

The recorded torque signals were low-pass filtered with a fourth-order, zero-lag Butterworth filter with a cut-off frequency of 10 Hz. For each participant, the highest peak torque obtained during the isometric MVC and the highest peak torque obtained during the isokinetic MVC (SI unit: N·m) were used as measures of maximal trunk extension strength. To enable statistical comparisons between groups, both torque values were normalised to each participant’s body mass (N·m·kg^− 1^). During the isometric and isokinetic submaximal tasks, torque steadiness was quantified as the amplitude of torque fluctuations, expressed in relative terms by the coefficient of variation of the torque signal (CoV = SD/mean × 100) over time. Both torque SD and torque_mean_ were expressed as percentages of the maximum torque obtained during the MVC contractions. The CoV of torque was calculated separately for each repetition, and the resulting values were averaged to obtain a single value for each torque level within each contraction condition (isometric or isokinetic). The same time window selected for the HDsEMG analysis was used for this calculation.

### Statistical analysis

Statistical analysis was performed using IBM SPSS 24.0 (IBM Corp., Armonk, NY, United States) and Jamovi 1.6.23 (The Jamovi project, Sydney, Australia). The Shapiro-Wilk test was used to check normality of the data. When normality was not assumed, nonparametric tests were applied. Independent t*-*tests were conducted to compare young and older participants in body mass and BMI, while the Mann-Whitney U test was used to assess group differences in height.

For the isometric contractions, the Mann-Whitney U test was used to assess group differences in baseline isometric MVC values (MVC_pre__ISO). The Wilcoxon signed-rank test was then applied to evaluate within-group changes between MVC_pre__ISO and post-task values (MVC_post__ISO) following the isometric submaximal contractions. Linear Mixed Models (LMMs)^51^ were applied to the RMS_norm_ values. In contrast, Generalised Linear Mixed Models (GLMMs)^51^ were used for the HDsEMG-torque coherence values (z-coherence), the centroid of coherence on both axis (coher_x and coher_y), and torque variables (torque_mean_ and torque CoV), as the residuals of these variables did not meet the assumption of normality.

For the isokinetic contractions, an independent t*-*test was conducted to compare young and older participants in baseline isokinetic MVC values (MVC_pre__DYN). Paired-samples t-tests were then performed to examine within-group differences between MVC_pre__DYN and post-task values (MVC_post__DYN) following the isokinetic submaximal contractions. LMMs were applied to RMS_norm_, coher_x, and coher_y, whereas GLMMs were used for z-coherence, torque_mean_ and torque CoV.

Group (young vs. older), Torque (25% vs. 50%), and Muscle (right LES vs. left LES) were included as fixed effects in all LMMs and GLMMs for both contraction types, with participant ID entered as a random intercept to account for within-subject variability. However, muscle side was not considered for torque-related variables, as torque values were obtained from the global output of both muscles. Sex was also added as a fixed effect in supplementary analyses to explore potential sex-related effects. The LMMs were fitted using restricted maximum likelihood estimation (REML), and the significance of fixed effects was assessed using Satterthwaite’s approximation for the degrees of freedom. For the GLMM, appropriate distributions and link functions were specified according to the data distribution.

For all analyses, the significance level (α) was set to 0.05. When multiple comparisons were performed, *p*-values were adjusted using the Holm-Bonferroni correction. The data are reported as mean ± Standard Deviation (SD) for parametric data and as median and interquartile range (IQR) for nonparametric data.

## Results

### Participants’ characteristics

No significant differences were found in body mass, height and BMI between young and older participants (*p* > 0.05). Descriptive characteristics of all participants are summarised in Table 1. Physical activity levels are summarised in Table 2, which presents the score ranges for each tertile (low, moderate and high physical activity) separately for each group, along with the percentage of participants in each tertile.

**Table 1:** Characteristics of all participants separated by group. *BMI* body mass index. *BBS* Berg Balance Scale. *MVC*_*pre*_*_ISO* baseline isometric maximal voluntary contraction. *MVC*_*pre*_*_DYN* baseline isokinetic maximal voluntary contraction. Data are presented as mean ± SD for all variables except for height and MVC_pre__ISO (median and IQR). The asterisk (*) indicates statistically significant differences between groups.

Characteristic	Elderly (*n* = 20; 12 F, 8 M)	Young (*n* = 20; 12 F, 8 M)	*p* value
Age (years)	74.80 ± 4.74	24.75 ± 5.00	–
Body mass (kg)	65.05 ± 11.56	64.15 ± 11.08	0.803
Height (m)	1.65 (0.24)	1.69 (0.14)	0.314
BMI (kg/m^2^)	23.06 ± 2.54	21.90 ± 2.69	0.169
BBS score	54.75 ± 1.33	–	–
MVC_pre__ISO (N⋅m⋅kg^− 1^)	2.08 (1.56)	2.89 (1.46)	0.013*
MVC_pre__DYN (N⋅m⋅kg^− 1^)	2.24 ± 0.74	2.99 ± 0.74	0.003*

**Table 2:** Physical activity levels for each group, showing score ranges for each tertile (low, moderate, and high physical activity), with participant percentages in brackets.

	Low physical activity level (tertile 1)	Moderate physical activity level (tertile 2)	High physical activity level (tertile 3)
Elderly	≤ 10.05 (35%)	10.06–18.74 (30%)	> 18.74 (35%)
Young	≤ 7.13 *(40%)*	7.14–8.68 *(25%)*	> 8.68 *(35%)*

### Isometric contractions

#### MVC

MVC_pre__ISO values were significantly lower in older compared to younger participants (*p* < 0.05) (Table 1). In addition, within each group, no significant differences were observed between MVC_pre__ISO and MVC_post__ISO (young: *p* = 1.000; elderly: *p* = 0.073), indicating that muscle fatigue did not occur.

#### Torque steadiness

**Fig. 3:**
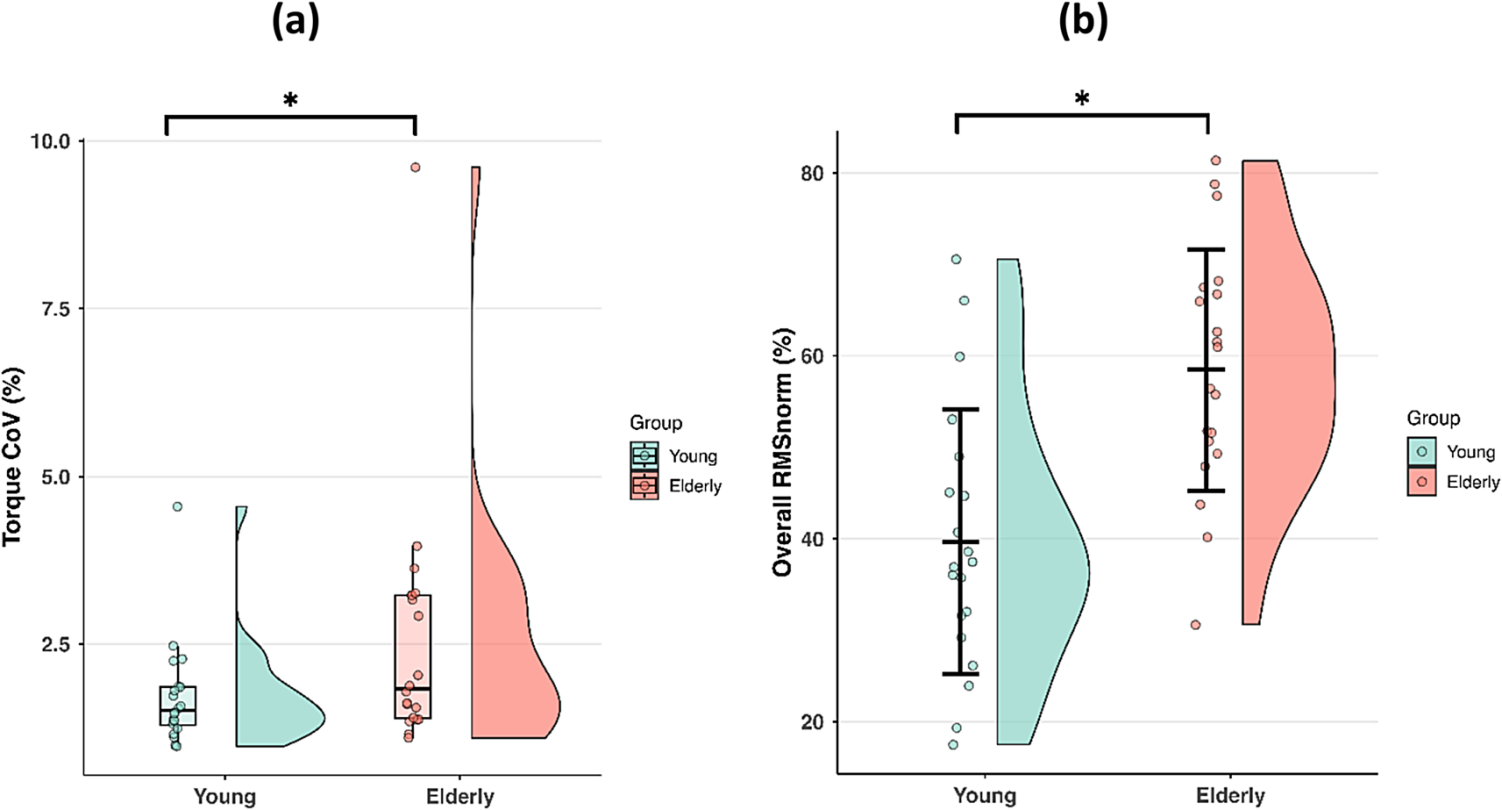
Torque CoV **(a)**, overall RMS_norm_ values **(b)** in young and elderly participants during the isometric trunk extension contractions. Median (IQR) for torque CoV. Mean ± SD for RMS_norm_ values. The distributions are illustrated using half-violin plots with embedded boxplots and individual data points for torque CoV, and mean ± SD plots with individual data points for overall RMS_norm_ values. Data are pooled across torque levels and muscles.* Main effect of Group.

A significant main effect of Group was found for torque CoV (X^2^ = 21.164; exp(B) = 1.318; *p* < 0.001), indicating overall higher CoV values in older individuals (Fig. 3a). Specifically, older adults exhibited 23.03% greater CoV relative to younger individuals. In addition, a significant main effect of Sex was observed (X^2^ = 13.981; exp(B) = 1.161; *p* < 0.001), with females exhibiting higher CoV values than males. Lastly, the exerted torque_mean_ at the two torque levels was similar between groups (X^2^ = 0.318; exp(B) = 1.010; *p* = 0.573). No significant interactions were found for either torque CoV or torque_mean_ (*p* > 0.05).

#### RMS_norm_

The fixed-effects omnibus test revealed a significant main effect of Group (F = 92.956; *p* < 0.001), with older participants exhibiting overall greater RMS_norm_ values than younger participants (Fig. 3b). A significant main effect of Torque was also found (F = 37.077; *p* < 0.001), with RMS_norm_ values being higher at 50% MVC compared to 25% MVC. In addition, a significant main effect of Sex was found (F = 12.985; *p* < 0.001), indicating higher values in females than in males. No significant interactions emerged (*p* > 0.05).

#### Coherence

**Fig. 4:**
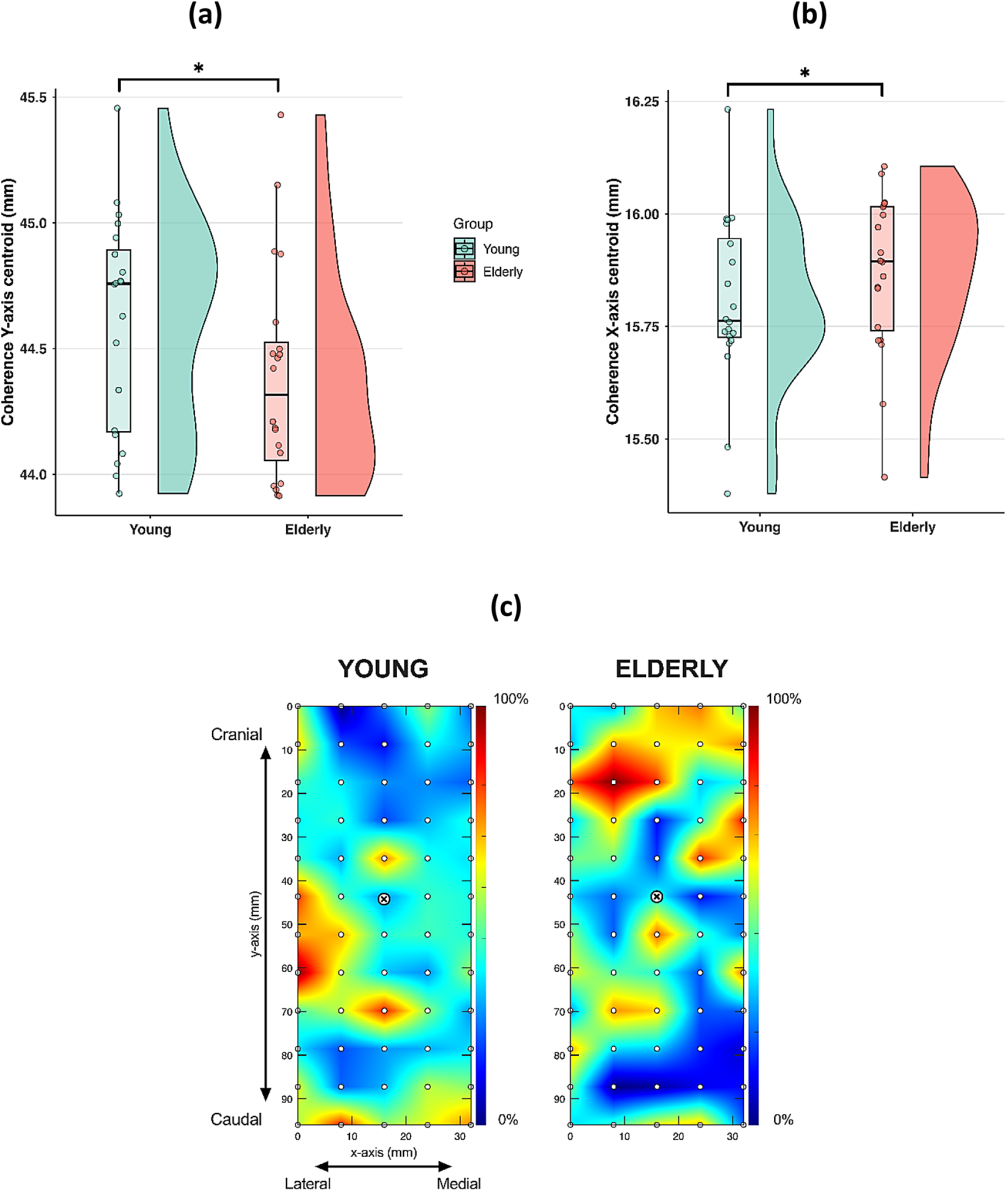
Centroid of HDsEMG-torque coherence along the y-axis **(a)** and along the x-axis **(b)** in young and elderly participants during the isometric trunk extension contractions. Median (IQR). The distributions are illustrated using half-violin plots with embedded boxplots and individual data points. Data are pooled across torque levels and muscles. * Main effect of Group. Representative topographical maps of coherence for one young and one older participant **(c)**. The white circle in the middle of the maps represents the centroid of coherence. The spine is located towards the medial side of the x-axis.

A significant main effect of Group on the centroid of HDsEMG-torque coherence along the y-axis was observed (X^2^ = 7.978; exp(B) = 0.995; *p* = 0.005), indicating that HDsEMG-torque coherence was higher in more cranial regions of the LES of older participants compared to younger individuals (Fig. 4a). A significant main effect of Group was also found for the centroid of coherence along the x-axis (X^2^ = 10.343; exp(B) = 1.000; *p* = 0.001), with older participants showing higher HDsEMG-torque coherence in more medial regions of the LES compared to younger individuals (Fig. 4b). No significant interactions were found for the centroid of coherence along either the x- or y-axis (*p* > 0.05). Topographical maps showing HDsEMG-torque coherence distribution for both groups are presented in Fig. 4c. Lastly, no significant main effects or interactions were found for z-coherence values (young: 1.62 (0.27); elderly: 1.60 (0.25); *p* > 0.05).

### Isokinetic contractions

#### MVC

MVC_pre__DYN values were significantly lower in older compared to younger participants (*p* < 0.05) (Table 1). Within each group, no significant differences were observed between MVC_pre__DYN and MVC_post__DYN (young: *p* = 0.131; elderly: *p* = 0.490), indicating that muscle fatigue did not occur.

#### Torque steadiness

**Fig. 5:**
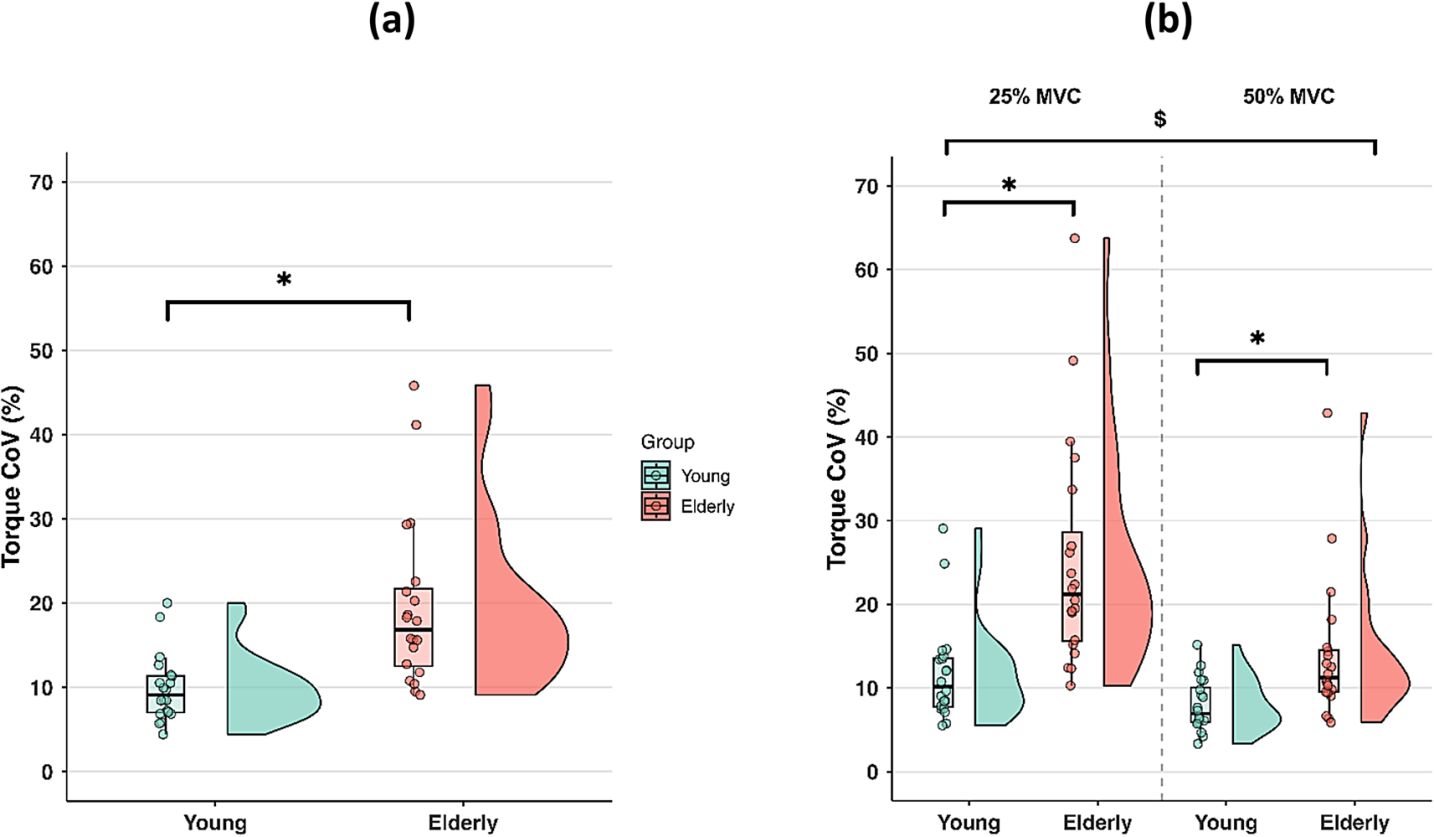
Overall torque CoV **(a)**, and torque CoV at 25% and 50% MVC **(b)** in young and elderly participants during the isokinetic trunk extension contractions. Median (IQR). The distributions are illustrated using half-violin plots with embedded boxplots and individual data points. Data are pooled across torque levels and muscles in figure **(a)**, while they are pooled across muscles only in figure **(b)**. * Main effect of Group. **$** Group x Torque interaction.

A significant main effect of Group was found for torque CoV (X^2^ = 89.470; exp(B) = 1.666; *p* < 0.001), indicating overall higher values in older individuals (Fig. 5a). Specifically, older adults exhibited 72.62% greater CoV relative to younger individuals. A significant Group x Torque interaction was also found (X^2^ = 7.290; exp(B) = 0.784; *p* = 0.007) (Fig. 5b). Post-hoc analyses revealed significantly greater CoV values in the older group compared to the younger group at both 25% and 50% MVC (*p* < 0.001). However, torque CoV was 107.84% higher at 25% MVC and 61.37% higher at 50% MVC in older individuals compared to the younger group, indicating that group differences were more pronounced at lower contraction intensities. Lastly, the exerted torque_mean_ at the two torque levels was similar between groups (X^2^ = 0.412; exp(B) = 1.035; *p* = 0.521).

#### RMS_norm_

**Fig. 6:**
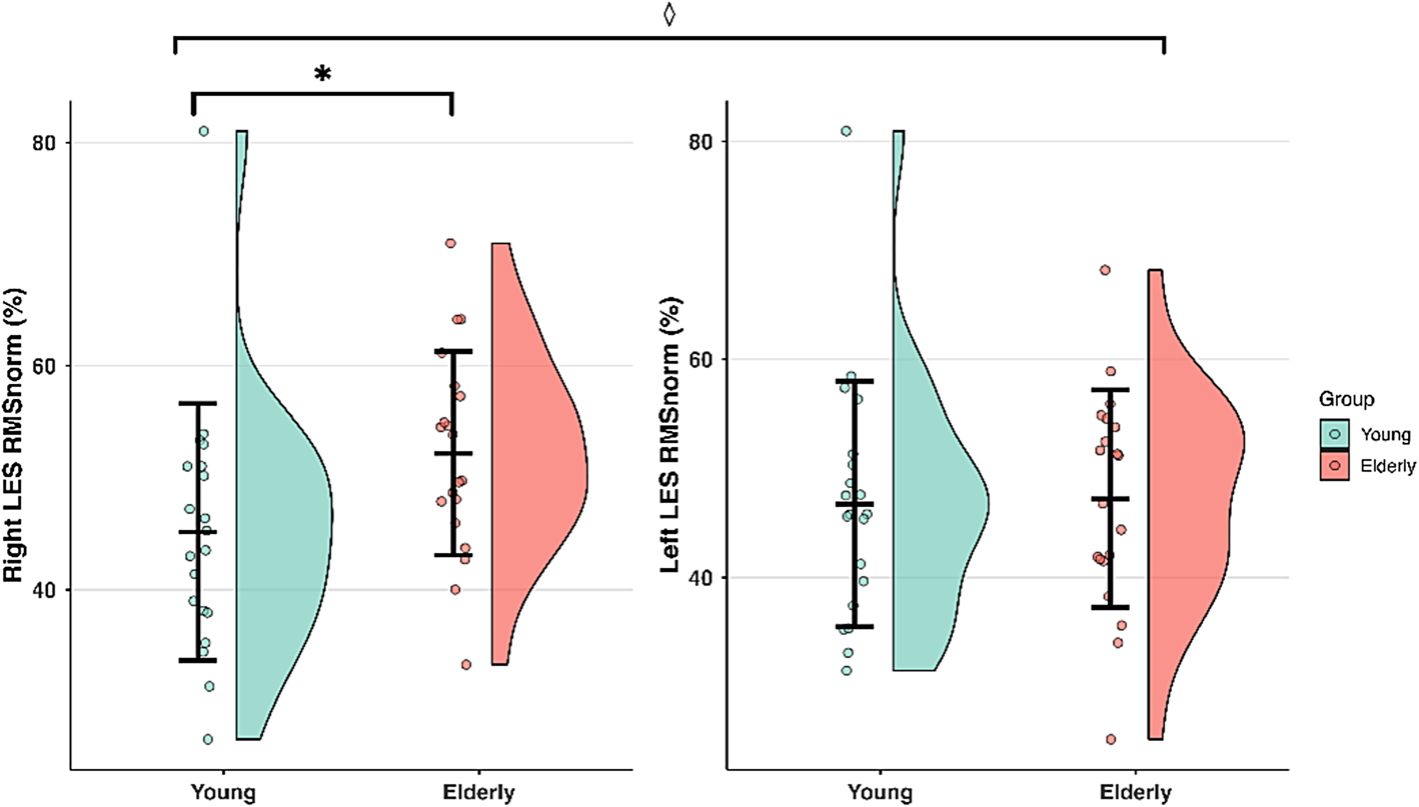
RMS_norm_ values in the right and left LES in young and elderly participants during the isokinetic trunk extension contractions. The distributions are illustrated using half-violin plots with embedded mean ± SD plots with individual data points. Data are pooled across torque levels. * Main effect of Group. ◊ Group x Muscle interaction.

A significant main effect of Group was observed for RMS_norm_ values (F = 8.151; *p* = 0.005), with older participants exhibiting overall higher RMS_norm_ values compared to younger participants. RMS_norm_ values also showed a significant main effect of Torque (F = 102.091; *p* < 0.001), being greater at 50% MVC than at 25% MVC. In addition, a significant Group x Muscle interaction was found (F = 5.163; *p* = 0.025) (Fig. 6). Post-hoc comparisons revealed that older participants showed significantly greater RMS_norm_ values in the right LES compared to the young group (*p* = 0.002), but not in the left LES (*p* > 0.05). A trend toward higher values in the right than left LES within the older group was also found (*p* = 0.065).

#### Coherence

**Fig. 7:**
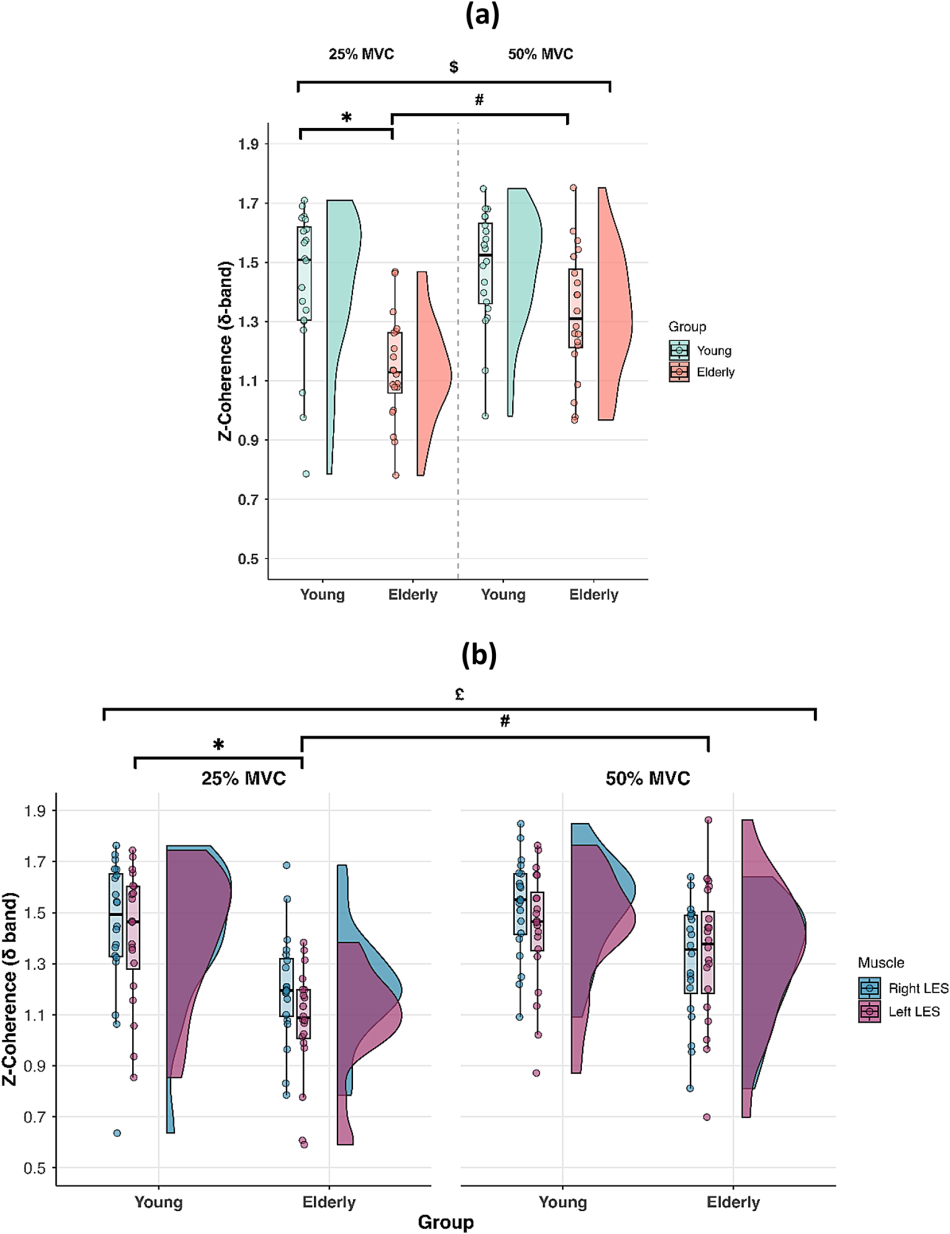
Z-coherence values at 25% and 50% MVC **(a)**, and z-coherence values in the right and left LES at each contraction intensity **(b)** in young and elderly participants during the isokinetic trunk extension contractions. Median (IQR). The distributions are illustrated using half-violin plots with embedded boxplots and individual data points. Data are pooled across muscles in figure **(a)**. * Main effect of Group. **#** Main effect of Torque. **$** Group x Torque interaction. **£** Group x Torque x Muscle interaction.

A significant main effect of Group was observed for z-coherence values (X^2^ = 25.819; exp(B) = 1.110; *p* < 0.001), with older participants showing overall lower coherence values compared to younger individuals. A main effect of Torque was also found (X^2^ = 17.728; exp(B) = 0.928; *p* < 0.001), indicating higher z-coherence values during the 50% MVC compared to the 25% MVC condition. The values at 25% and 50% MVC were 1.30 (0.45) and 1.44 (0.34), respectively. In addition, a significant Group x Torque interaction was detected (X^2^ = 8.148; exp(B) = 0.904; *p* = 0.004) (Fig. 7a). Post-hoc analyses revealed a significant difference between groups at 25% MVC (*p* < 0.001), but not at 50% MVC (*p* = 0.110), indicating that older participants exhibited lower z-coherence values than younger participants only at the lower intensity level. Moreover, higher z-coherence values at 50% MVC compared to 25% MVC were observed in older participants (*p* < 0.001), but not in younger individuals (*p* = 0.503). Lastly, a significant Group x Torque x Muscle interaction was found (X^2^ = 4.093; exp(B) = 0.866; *p* = 0.043) (Fig. 7b). Post-hoc comparisons showed a significant increase in z-coherence values from 25% to 50% MVC in the left LES of older participants (*p* < 0.001), whereas no change was observed in the right LES (*p* = 1.000). In younger participants, z-coherence values did not differ between contraction levels for either muscle (*p* > 0.05). In addition, a significant difference between groups was observed for left LES at 25% MVC (*p* < 0.001), with older participants showing lower z-coherence values. A trend toward a similar difference was found for the right LES (*p* = 0.054). Instead, no significant differences emerged at 50% MVC (*p* > 0.05).

Regarding the centroid of coherence, no significant main effects or interactions were found along the y-axis (*p* > 0.05), while a significant main effect of Muscle was observed along the x-axis (F = 85.681; *p* < 0.001), with the left LES exhibiting coherence that was overall more medial compared to the right LES.

## Discussion

The present study investigated the relationship between LES HDsEMG amplitude and torque fluctuations during isometric and isokinetic concentric trunk extension contractions in young and older adults, using coherence analysis (0–5 Hz). In addition, regional differences in HDsEMG-torque coherence of the LES were characterised in both groups. Consistent with our hypothesis, older adults exhibited greater torque CoV during both isometric and isokinetic contractions, indicating reduced control of trunk extensor muscle force. Notably, the age-related difference was larger during isokinetic contractions and, within these, was most pronounced at the lower intensity (25% MVC). Contrary to our expectations, the older group exhibited a weaker correlation between HDsEMG and torque (i.e., lower coherence values) only during isokinetic contractions, whereas alterations in the regional distribution of LES HDsEMG-torque coherence were evident exclusively during isometric contractions.

### Age-related reductions in maximal torque

It is well-documented that isometric maximal strength/torque of the trunk extensor muscles declines with age^30,31,52,53^. In contrast, fewer studies have examined age-related differences in trunk strength during isokinetic contractions. Existing evidence indicates that older adults exhibit lower peak torque values than younger individuals across multiple angular velocities, ranging from slow (15°/s) to fast (180°/s) contraction speeds^54,55^. Accordingly, the present study also observed reduced maximal isokinetic torque in older adults, assessed at a lower angular velocity (10°/s). Therefore, our findings extend previous evidence by demonstrating that the age-related decline in trunk extensor strength is also present under slower contraction conditions. A recent study has shown that this decline may not be primarily attributed to age-related atrophy and fat infiltration of the paravertebral muscles, but rather to neuromuscular mechanisms such as a decline in the neural drive to the muscles or an age-related remodeling of muscle phenotype^54^.

Moreover, neither isometric nor isokinetic maximal muscle torque decreased following the respective submaximal contraction tasks in either group. This suggests that no significant muscle fatigue was induced during the tasks, which is important as fatigue could have represented a potential source of bias in both the mechanical (i.e., muscle force control) and physiological assessments^56^.

### Impaired torque steadiness in older adults

The reduced torque steadiness (i.e., greater torque fluctuations) observed in older individuals in the present study is consistent with a meta-analysis demonstrating a large overall effect of age on force steadiness across both upper and lower limb muscles^57^. Although less frequently investigated, similar age-related impairments have been reported in the trunk extensors^25,30,31^. In particular, previous studies have shown an age-related increase in force fluctuations of the trunk extensor muscles during a fatiguing task performed at 30% of maximal voluntary isometric force^30^ and during a 15-s steadiness task at 10% of peak torque^31^. Similarly, trunk position and force sense have been shown to be altered in older individuals with hyperkyphosis^32^. However, in contrast to the present findings, no differences in force fluctuations between young and older participants were reported at 50% of peak torque^31^. This discrepancy may be explained by different factors, such as the younger mean age of the older group in that study (69.22 ± 4.10 years) compared with the present sample, as well as differences in the experimental setup. In addition, our findings are in accordance with previous studies on large proximal muscles, such as the knee extensors, showing that greater force fluctuations are still evident up to 50% MVC, although the effect of ageing is greater at lower contraction intensities^57^. However, all the studies mentioned so far have focused on isometric contractions of the trunk extensor muscles, whereas muscle force control during dynamic contractions has been investigated only in individuals with chronic low back pain^24^. Thus, to the best of the authors’ knowledge, the present study is the first to examine force control of the trunk extensor muscles during dynamic contractions in older adults.

Notably, we found that the difference in force steadiness between the two groups was greater during isokinetic contractions compared to isometric contractions. This finding aligns with some previous studies reporting larger age-related increases in force fluctuations during sinusoidal, concentric, and eccentric tasks than during isometric contractions^7,8^. Such differences may have meaningful functional implications, as dynamic contractions more closely reflect the movements performed during everyday activities. Moreover, during the isokinetic contractions, the difference in force steadiness between groups was more pronounced at 25% MVC than at 50% MVC, indicating that age-related impairments in force control were greater at lower contraction intensities. This finding is consistent with previous evidence showing reduced stability in older adults particularly at lower target forces, such as during slow anisometric contractions of the first dorsal interosseous muscle at lighter loads^6^ and during low-intensity isokinetic contractions of the quadriceps^58^. This age-related increase in force fluctuations at predominantly lower contraction intensities is particularly relevant, as most activities of daily living, especially those commonly performed by older adults, typically require forces of up to approximately 20% MVC^12^.

It should also be noted that a potential mechanical contributor to force fluctuations during the isokinetic contractions in both groups is the effect of muscle-tendon length on torque production capacity^59,60^. In particular, torque production of the hip and trunk extensors varies with hip angle due to the force-length relationship^60,61^. Therefore, changes in muscle-tendon length across the range of motion may have contributed to the challenge of controlling torque during the isokinetic contractions at specific joint angles in both groups. Lastly, the greater force fluctuations observed in females during isometric contractions, regardless of age group, are consistent with previous evidence and may be attributable to sex-specific differences in neuromuscular control strategies^62,63^.

### Age-related alterations in HDsEMG-torque coherence

Significant differences in HDsEMG-torque coherence values were observed between groups during the isokinetic trunk extension contractions, whereas no differences were observed during the isometric contractions. This finding is somewhat surprising, as we expected lower HDsEMG-torque coherence in older adults during both isometric and isokinetic tasks, given that they exhibited greater torque fluctuations than younger participants in both conditions. Indeed, lower coherence between HDsEMG activity and torque would reflect reduced coupling between the neural input to the motor-unit pool and the mechanical output, which can, in turn, alter force stability^24^.

The decrease in coherence between oscillations in LES activity and torque generation observed in older adults during isokinetic contractions was primarily driven by a reduction in coherence at the lower contraction intensity (25% MVC). At this intensity, the reduction in HDsEMG-torque coherence was mainly attributable to a muscle-specific difference rather than a uniform bilateral reduction, with the left LES contributing less to torque. However, as shown in Fig. 7b, a similar trend was also observed for the right LES. The emergence of this asymmetry at the lower contraction intensity may reflect age-related postural adaptations and/or compensatory motor strategies (e.g., lateral flexion or trunk rotation) that become apparent during dynamic tasks performed at lower force levels, which typically require greater precision and coordination^31^. Under these conditions, older adults may have reduced the contribution of one side of the trunk extensors to the resultant torque and redistributed the load across multiple synergistic muscles to generate the required torque. This may have led to increased force variability, as recruiting additional synergistic muscles can amplify force fluctuations due to each muscle operating along slightly different action directions^64^. Additionally, greater coactivation in older adults cannot be excluded, which may have further influenced coherence values and torque generation, as alternating activation of agonist and antagonist muscles can increase fluctuations in force and acceleration^65^. Although the reduction in HDsEMG-torque coherence reached statistical significance only for the left LES, the presence of a similar trend in the right LES suggests that the observed effect may not be strictly unilateral. Importantly, the contraction intensity at which the reduction in HDsEMG-torque coherence emerged also corresponded to the largest impairment in force steadiness in older adults, with torque CoV being 107.84% higher than in younger participants. In contrast, no differences in HDsEMG-torque coherence were observed at 50% MVC, although older adults still exhibited reduced force steadiness. At this higher intensity, it is plausible that older adults were required to activate the LES more, contributing to torque generation similarly to younger individuals. Nevertheless, they may have continued to rely more on synergistic muscles than younger participants, thereby resulting in greater force fluctuations, although to a lesser extent than at 25% MVC. Collectively, these results suggest that previous evidence of age-related reductions in neural-mechanical coupling, particularly at lower contraction intensities^6,65^, also applies to the trunk extensor muscles, at least during isokinetic contractions.

In contrast, no differences in HDsEMG-torque coherence values between groups were observed during the isometric trunk extension contractions. During these contractions, it is possible that older adults were unable to use the compensatory strategies likely adopted during the isokinetic contractions due to the more constrained position. Nevertheless, they were still less stable than the younger individuals. As reported by Enoka et al.^65^, during simpler tasks such as isometric contractions, the properties of individual motor units within a single muscle play a more dominant role in force control, whereas in more complex movements (e.g., isokinetic contractions), force fluctuations depend more on the distribution of activity among multiple muscles. Therefore, it is possible that, although the LES contributes similarly to torque generation in both young and older individuals, inherent alterations in the common synaptic input to the motor unit pool (i.e., in the oscillatory components of this input) in older adults may underlie their reduced force steadiness^17^. However, to study these mechanisms, motor unit decomposition analysis would be necessary. Unfortunately, this remains challenging in the trunk muscles due to several factors, such as the complex spinal anatomy (e.g., the presence of multiple muscle layers and thoracolumbar fascia) and the volume conductor characteristics of the lower lumbar region (e.g., thick subcutaneous tissue layer)^66^. Although HDsEMG-torque coherence values were similar between groups, differences emerged in their spatial distribution, with older adults showing a more cranial and medial coherence pattern within the LES. The greater contribution to torque from the more cranial regions in older adults is consistent with our previous study^30^ and may reflect a compensatory mechanism aimed at redistributing the load toward the upper lumbar regions, as the L4-L5 segments commonly exhibit greater degeneration with ageing^67^. Instead, the more medial coherence may be related to greater recruitment of the superficial multifidus muscles located closer to the spine, possibly to help stabilise the trunk, particularly in the presence of the more flexed posture commonly associated with ageing^68^. This neuromuscular strategy adopted by older individuals may contribute to their reduced torque steadiness, as a more flexed posture and greater recruitment of the upper lumbar vertebrae can limit proper activation of the LES^24^.

### Age-related differences in RMS_norm_

Overall greater activation (i.e., higher RMS_norm_ values) of the LES was observed in older adults during both isometric and isokinetic submaximal contractions. This finding is consistent with previous studies reporting increased muscle activation in older adults compared to younger individuals to generate the same amount of force during force steadiness tasks, along with greater force fluctuations^6,69,70^. However, during the isokinetic trunk extensions, this difference in muscle activation was primarily driven by greater activation of the right LES muscle in the older group, whereas no such difference between muscles was found during isometric contractions. Although RMS and coherence analyses assess different aspects of neuromuscular function, the observed difference in activation between muscles further supports the presence of potential asymmetries between the bilateral LES in older adults during dynamic contractions. This finding may reflect postural adaptations associated with ageing that are not evident during isometric contractions but become apparent during dynamic tasks, which better reveal underlying motor impairments. In line with this interpretation, some previous studies have reported age-related deficits or asymmetries emerging primarily during dynamic or challenging tasks, while remaining small or absent during static or isometric tests^71–73^. Lastly, regardless of age group, females demonstrated higher RMS_norm_ values than males during isometric contractions, which may be related to sex differences in muscle fibres activation (e.g., higher motor unit discharge rate) to generate the same relative torque^74^.

### Practical applications

From a practical perspective, these findings suggest that interventions for older adults should address not only trunk extensor strength but also force control, particularly during dynamic tasks. The greater impairments observed during isokinetic contractions indicate that dynamic conditions, especially low-intensity tasks that typically require greater precision and coordination, may represent priority targets for exercise interventions. In this context, training approaches that provide real-time feedback, such as force-feedback or HDsEMG-based biofeedback, could help older adults restore optimal neuromuscular activation patterns and enhance motor control^75,76^. Importantly, these findings also have preventive relevance, as research in healthy older adults can inform exercise programs aimed at maintaining functional mobility and reducing the risk of musculoskeletal conditions such as chronic low back pain, which is highly prevalent with ageing^77^.

### Methodological considerations

A strength of this study is the use of HDsEMG, which enables the assessment of muscle activity over a larger area and provides higher spatial sampling resolution than traditional bipolar sEMG. In addition, the bilateral placement of electrode grids on the LES allows for a more comprehensive evaluation of lower back muscle activity. Another strength is the relatively advanced age of our older group, which allows for a clearer characterisation of age-related neuromuscular adaptations. Despite these strengths, some limitations must be acknowledged. First, the sample consisted of healthy, well-functioning older adults, which limits the generalisability of the findings to other populations, such as frail older individuals. However, as this is among the few studies to have examined force control of the trunk extensor muscles in the context of ageing, it provides a good basis for future research involving populations at greater risk. In addition, the present study did not assess potential compensatory strategies, such as the activation of synergistic muscles (e.g., hip extensors) or antagonist co-activation, which may have contributed to task performance. Lastly, we acknowledge that the rectified sEMG provides only an indirect and coarse estimate of the neural drive to the muscles, as it can be influenced by several physiological and methodological factors. A more accurate estimation of the neural drive can be achieved using HDsEMG decomposition analysis, which allows the identification of specific motor unit discharge patterns. However, as previously mentioned, this approach remains challenging in the trunk muscles.

## Conclusions

Force control of the trunk extensor muscles is impaired in older individuals. Specifically, we uniquely demonstrated that age-related impairments in torque steadiness are more pronounced during isokinetic than isometric trunk extension contractions, with the greatest deficits observed at the lower contraction intensity (25% MVC). Moreover, distinct neuromuscular patterns emerged depending on contraction type: older adults exhibited reduced HDsEMG-torque coherence magnitude during isokinetic contractions and an altered spatial distribution of coherence during isometric contractions. Overall, these findings contribute to the existing body of knowledge on muscle force control (i.e., force/torque steadiness) in older adults and extends the current understanding to the trunk extensor muscles.

## Data Availability

Any materials related to this study are available upon reasonable request by contacting the lead author, Martina Parrella.
